# Arboviruses and the eye

**DOI:** 10.1186/s40942-016-0057-4

**Published:** 2017-02-01

**Authors:** Gabriel Costa de Andrade, Camila V. Ventura, Paulo Augusto de Arruda Mello Filho, Maurício Maia, Silvana Vianello, Eduardo Büchele Rodrigues

**Affiliations:** Department of Ophthalmology, Federal University of São Paulo - Paulista Medical School, Rua Botucatu, 821, 1st Floor, São Paulo, SP 04023-062 Brazil

**Keywords:** Arbovirus, Arboviral disease, Dengue virus, Chikungunya virus, Zika virus, Ocular manifestations, Retinal manifestations, Brazil

## Abstract

Arthropod-borne viruses, or arboviruses, are viruses that are transmitted through the bites of mosquitoes and ticks. There are numerous arboviruses throughout the world capable of causing human disease spanning different viral families and genera. Recently, dengue, chikungunya, and zika viruses have emerged as increasingly important arboviruses that can cause human disease, however no specific treatment or vaccine is available for them. In addition, ocular manifestations of these diseases have become more prevalent over the past few years. This review highlights the current understanding on the pathogenesis, systemic changes and ocular findings, emphasizing the retinal manifestations related to dengue, chikungunya, and zika viruses.

## Background

Arboviral diseases are entities that are transmitted to humans by arthropods such as mosquitoes and ticks. The first two letters of the words arthropod’ and borne, make up the ‘arbo’ that now designates this group of viruses as arthropod-borne [1]. The arboviruses include a wide variety of RNA virus including the flaviviruses (genus Flavivirus, one of three genera in the family Flaviviridae) and the alphaviruses (genus Alphavirus, one of two genera in the family Togaviridae). Both dengue fever virus (DFV) and zika virus (ZIKV) are flaviviruses whereas chikungunya virus (CHIKV) belongs to the alphavirus familiae [1].

The global expansion of these arboviruses was preceded by the global spread of their vectors. *Aedes aegypti* was originated in Africa, from where it subsequently spread globally to tropical and sub-tropical regions of the world. *Aedes albopictus*, originally a zoophilic forest specie from Asia, spread to islands in the Indian and Pacific Oceans. During the 1980s it rapidly expanded its range to Europe, the United States and Brazil [1]. Today both *A. aegypti* and *A. albopictus* are present in most Asian cities and large parts of the Americas. *A. aegypti* feed almost exclusively on humans in daylight hours and typically rest indoors. In contrast *A. albopictus* is usually exophagic and bites humans and animals opportunistically but has also been shown to exhibit strongly anthropophilic behavior similar to *A. aegypti* in specific contexts. Only females bite to obtain blood in order to lay eggs [1].

The illnesses caused by the arbovirus have very similar/overlapping clinical presentation with prominent fever, headache, rash, myalgia and arthralgia. In fact, serologic surveys have demonstrated that outbreaks attributed to dengue in the past have actually turned out to be CHIKV or ZIKV infections. DFV and CHIKV are genetically distant relatives, however ZIKV is closely related to dengue and often cross reacts on DFV serology or on some rapid assays that are available at some posts [1].

DFV and ZIKV are asymptomatic in approximately 75% of infections, in contrast to CHIKV where patients are symptomatic in 75–95% of infections [1]. DFV is considered as the most serious of these infections, because of the rare but potentially severe dengue shock syndrome” that can lead to death. Both CHIKV and ZIKV related deaths are usually associated with Guillain–Barré syndrome [2] although recently ten death cases of ZIKV infection unrelated to the Guillain–Barré syndrome have been confirmed in adults [2].

The three related arboviruses can cause ocular manifestations that may range in DFV from hyposphagma to maculopathy [3, 4]. In both ZIKV and CHIKV there is a more common involvement of the anterior segment including conjunctivitis, uveitis and increased intra-ocular pressure (IOP). However, vasculitis and retinal hemorrhages can also occur [5–7]. Furthermore, two recently published Brazilian studies have described microcephaly and retinal lesions after presumed ZIKV intrauterine infection [8, 9].

The purpose of this review is to provide an overview of most prevalent arboviruses in Brazil (DFV, ZIKV, CHIKV) regarding the main features, physiopathology, and ocular involvement (Table 1).

**Table 1:** Main emerging arboviruses in Brazil

Family	Genus	Virus	Main clinical features
Flaviviridae	Flavivirus	Dengue	Break-bone fever, dengue hemorrhagic fever and shock syndrome
		Zika	Mild fever, maculopapular rash
Togaviridae	Alphavirus	Chikungunya	Fever, arthralgia, arthritis

## Dengue fever

Dengue fever (DFV) is the most common mosquito borne viral disease in humans, and has become a major health concern due to recent increased incidence [10, 11]. *A. aegypti* mosquito is the main vector responsible for the transmission of this disease, which is predominant in tropical weather regions. There are four proven serotypes of DFV: DENV1, DENV2, DENV3 and DENV4, and the distinction between them occurred in 1940 and 1956 [10]. Each year in Brazil, one serotype is responsible for most cases during the epidemics [11].

The disease has a large clinical spectrum and reports of ocular manifestations had been recently published with complications ranging from mild blurring of vision to significant morbidity with severe visual impairment (Table 2).

**Table 2:** Systemic manifestations of Dengue, Zika and Chikungunya

Symptoms	Dengue	Zika	Chikungunya
Fever	High	Mild or absent	High
Pain	Muscle, joints, head and behind the eyes	Mild joint pain and edema	Severe joint pain and edema
Skin rash	Not associated with itchiness	Associated with itchiness	Associated with itchiness
Complications	Multiple organ failure: lungs, heart, liver, kidneys, central nervous system	Neurologic complications: muscle weakness, Guillan Barré syndrome	Chronic joint pain

### Physiopathology

After the inoculation, the virus first infects skin dendritic cells, where occurs the replication and virus migration to the lymph nodes until reach the bloodstream to trigger acute febrile phase. This phase lasts from 3 to 5 days. The viruses reach many organs, mainly the most perfused. After the exposure to the virus, T cells release interferon that suppress the bone marrow resulting in the release of cytokines and in the decrease of thrombocytes or platelets [10]. There are two major theories that can explain the severe cases associated with hemorrhagic disease: (a) Reinfection for a different serotype (non-neutralized virus opsonized form immunocomplexes), leading to an increase of vascular capillarity and in the emergence of more virulent strains.; (b) The thrombocytopenia is further complicated by the fall of coagulation factors, explained by liver damage, as a result from hepatocytes viral.

Dengue infection can affect the retina microcirculation either by direct viral infection or activation of inflammation trough an immune-mediated reaction [10].

Some ophthalmologic abnormalities have been described in patients who had DFV. The pathogenesis of lesions appears to be the same as the clinical disease: hemoconcentration, vasculitis, and coagulation disorders (Table 3) [12].

**Table 3:** Ophtalmologic manifestations of Dengue, Zika and Chikungunya

	Dengue	Zika	Chikungunya
Conjuntivitis	No	Yes	Yes
Uveitis	Mild and rare	Moderate and eventual	Moderate and eventual
Posterior segment findings	Choroiditis, retinitis, macular edema, neuroretinitis and optic disc neuritis, acute macular neuroretinopathy	Macular pigment mottling, macular chorioretinal atrophy, optic nerve hypoplasia, increased cup-to-disc ratio	Choroiditis, retinitis, neuroretinitis and optic disc neuritis

### Systemic manifestations

Dengue fever causes a variety of clinical symptoms such as severe headaches, fever, and myalgia (13), as well as arthralgias, nausea, vomiting and cutaneous rash (13, 18). The clinical picture is characterized by an abrupt onset of fever after 2–7 days of incubation period, with temperature reaching [13].

In cases with severe and repeated infection, hemorrhagic DFV may develop due to exacerbation of the immune response, leading to hypotension and dengue shock syndrome, thrombocytopenia and in bleeding manifestations (13, 18).

### Ocular disease

Ocular manifestations with visual impairment are uncommon in DFV but have been more frequently described in the literature over the last few years [10, 13]. Blurring of vision was the most common symptom, followed by scotoma [13]. The most common sign is hyposphagma (37% of the cases), followed by, in rare cases, (anterior?) uveitis and increased intraocular pressure [4].

Most patients with dengue-related ophthalmic complications recover spontaneously without any treatment [4]. Patients with severe visual loss or bilateral involvement can be treated with systemic steroids. Prognosis of dengue-related ophthalmic complications is favorable and, according to previous reports, almost all patients show improvement in visual acuity and complete resolution of the ophthalmic complications [3, 4].

### Retinal manifestations

The majority of ocular changes in DFV are in the vascular system of the posterior segment. Maculopathy is the main retinal manifestation (affecting up to 10% of the cases) and is generally bilateral (73%) and characterized by vasculitis and hemorrhages [3, 4].

**Fig. 1:**
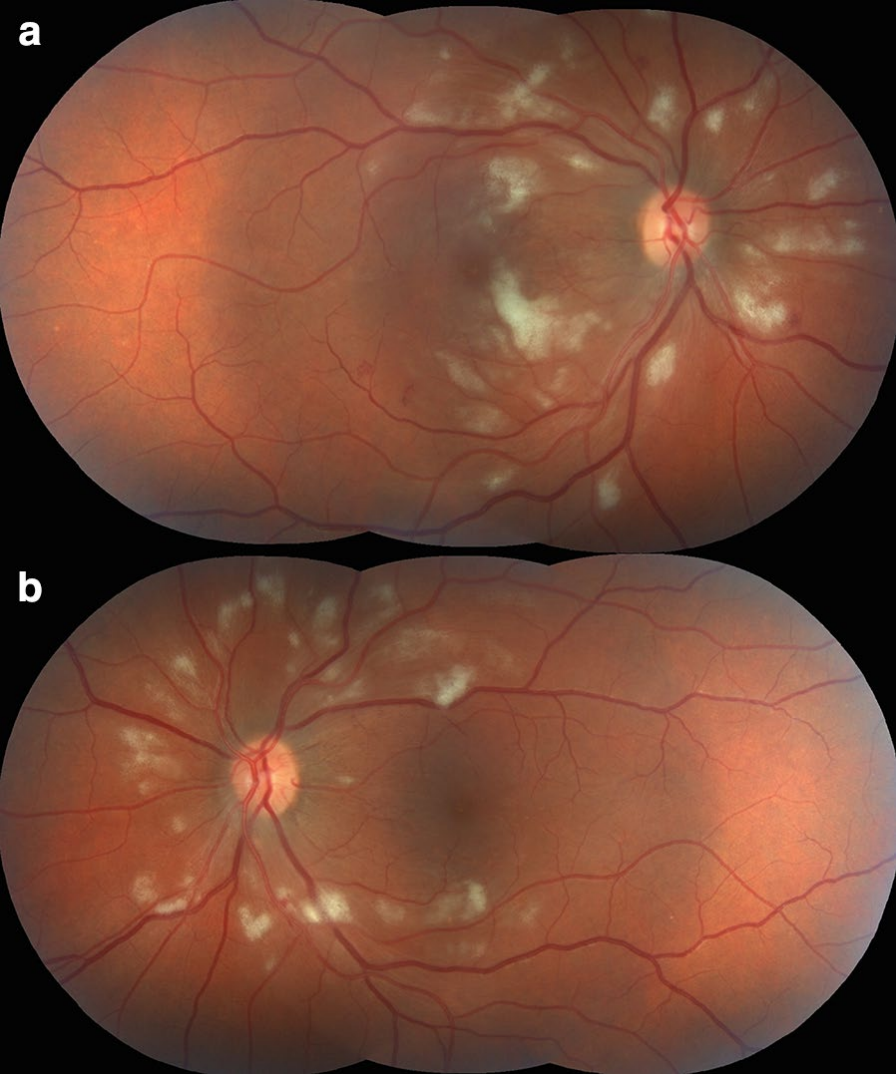
Fundus photographs of the right (**a**) and left (**b**) eyes of a patient with presumed retinitis secondary to Dengue virus infection reveals cotton-wool spots and retinal hemorrhage

Other posterior segment manifestations reporte include retinal venular widening, higher retinal vascular dimension, vascular sheathing, tortuous vessels, acute macular neuroretinopathy, intraretinal macular, peripheric or peripapillary hemorrhages, retinal edema (macular and diffuse), cotton wool spots (Fig. 1), Roth’s spot, serous retinal detachment, choroidal effusions, choroidal neovascularization, optic disc swelling and optic disc neuropathy [5, 13–17].

## Zika virus

Zika Virus (ZIKV), a Flavivirus, was first isolated in *Rhesus* monkeys from the Zika forest, located in Kampala, Uganda, in 1947 [18]. Five years later, it was isolated in Africans for the first time [19]. The virus then migrated to the Asian continent during the 40’s as a different strain from the one found in Africa [20]. In the last two decades, the Asian strain has been causing outbreaks outside Asia in other countries such as Micronesia, French Polynesia, and Easter Island in Chile [21–23]. However, the most recent and widest ZIKV outbreak in history ever reported started in May 2015 in the northeast of Brazil [24, 25]. It is estimated that between 0.4 and 1.3 million people have been infected by ZIKV in 2015 [26].

### Physiopathology

ZIKV is a mosquito-borne infection, mainly transmitted in Americas by *A. aegypti*, the same vector that transmits DFV and CHIKV [24]. Additionally, there are also reports of ZIKV infection following sexual, perinatal and blood transfusion. However there is still no explanation for the mechanism of these transmissions [25–27].

ZIKV can also lead to severe congenital malformations in newborns whose mothers were infected during pregnancy, especially in the first trimester of pregnancy.

However, the exact mechanism by which ZIKV can cause the congenital Zika syndrome, are still unknown. Studies indicate that the virus is able to evade the normal immunoprotective response of the placenta [28]. Two hypotheses regarding the placental role have been hypothesized: one is that the virus has neurotropic properties and, via the placenta directly affects and damages the brain development. The other suggested mechanism is that the placental response to the virus would be the main cause of central nervous system impairment, since the virus can cause an interruption of the placental outer layer synthesis, and leading to the congenital Zika syndrome [28].

### Systemic manifestations

Only 20% of patients infected with ZIKV are symptomatic. The symptoms include fever, headache, maculopapular rash, arthralgia, and conjunctivitis, which usually lasts for 1 week [21]. Severe disease caused by ZIKV, were recently described in patients from Brazil and French Polynesia, as the Guillain-Barré syndrome and other neurological manifestations in patients infected by the virus [24].

Furthermore, congenital Zika syndrome was recently described as another complication of ZIKV when the infection occurs during pregnancy, especially in the first trimester of pregnancy [24, 29, 30]. Although microcephaly is the major finding in these newborns, recent publications have described other malformations associated to ZIKV congenital infection including hearing loss, limb anomalies and ocular findings [9, 10, 31, 32]. Therefore, a new terminology has been given to this clinical condition, Congenital Zika Syndrome (CZS) [32].

However, since the current evidence of ZIKV infection relies on the molecular detection of viral RNA, which is positive only in a brief period of viraemia and the available serology to identify identifying IgM and IgG specific for Zika antibodies are not reliable due to cross-reactivity among other flaviviruses, further studies are necessary to better elucidate these findings and its correlation to ZIKV [33].

### Anterior ocular manifestations

Recent reports showed a mild disease in adults with acute infection, which can include anterior uveitis and non-purulent conjunctivitis [34]. The treatment of anterior uveitis related to ZIKV evolves topical corticosteroids has a benign prognosis [34]. In a study conducted in Salvador-BA, Brazil, patients with ZIKV-related congenital Zika syndrome showed anterior segment findings as iris coloboma and lens subluxation [31].

### Retinal manifestations

**Fig. 2:**
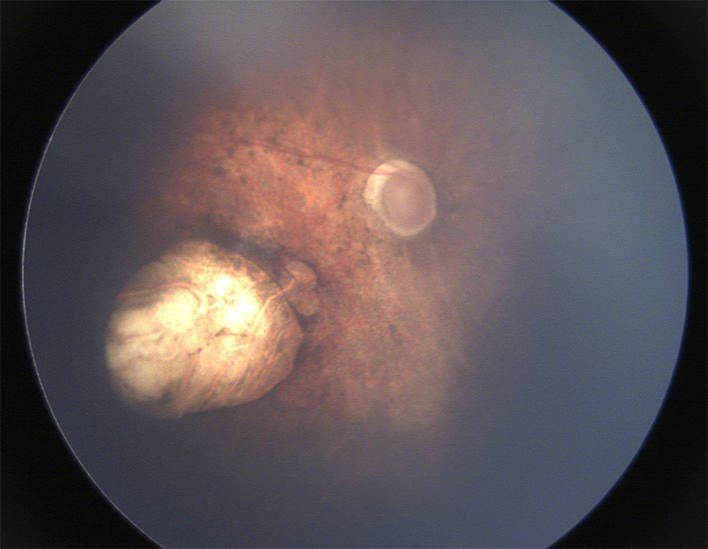
Wide-angle fundus image (Retcam^®^) of the right eye of an infant with presumed Zika virus congenital infection showing sharply demarcated chorioretinal scarring with gross pigment mottling on the macula

In Brazil, investigators of Recife and Salvador have reported the ocular abnormalities in infants clinically diagnosed with ZIKV-related microcephaly, which included retinal and optic disc findings that vary in a very broad spectrum [9, 10, 31]. These findings include gross macular pigment mottling, macular chorioretinal atrophy (Fig. 2), optic nerve hypoplasia and increased cup-to-disc ratio [9, 10, 31].

## Chikungunya

Chikungunya fever (CHIKV) is an emerging mosquito-borne disease caused by an *alphaviru*s, from the *Togaviridae* family [32]. The vectors are mosquitoes of the *Aedes* genus, the most common being *A. aegypti* in Americas and *A. Albopictus* in Asia, the same species involved in the transmission of DFV and ZIKV. CHIKV has been identified in over 60 countries in Asia, Africa, Europe and the Americas [32]. The virus is transmitted from human to human by the bites of infected female mosquitoes. After the bite of an infected mosquito, onset of illness occurs usually between 4 and 8 days but can range from 2 to 12 days [32].

### Physiopathology

The pathophysiology of human CHIKV infection is not clear. Studies on mice models have confirmed involvement of connective tissue (especially in epimysium), muscle, joint, skin fibroblasts, and even in the central nervous system (CNS) but not in fetal or placental tissues [33, 35]. CHIKV was also detected in the biopsies of muscles, joints, and dermis of infected human patients.

The persistence of CHIKV and/or CHIKV-encoded components in deep sanctuaries could play a role in chronic symptoms and biological changes in human infection, such as the slow disappearance of anti-CHIKV IgM in the serum of patients with long-lasting arthralgia [35, 36]. These data are consistent with the hypothesis of a persistent viral challenge with a plausible direct contribution to synovial tissue damage. It also supports the use of immunomodulation with disease-modifying drugs for the most affected chronic patients, despite a theoretical risk of viral reactivation [33, 35].

### Systemic manifestations

As with several other mosquito-borne alphaviruses, CHIKV causes a fever-rash-arthralgia syndrome in humans. The name “Chikungunya” derives from the debilitating joint pain noted by local populations during an outbreak in 1952–1953 in what is now Tanzania [37]. The local word means “that which bends up” and the name was given as a result of the stooped posture that resulted from the pain of the disease [38]. Acute infection lasts for 1–10 days and is characterized by an abrupt onset of fever, headache, fatigue, nausea, vomiting, rash, myalgia, and severe arthralgia. Joint pains may persist for months to years in some patients (published studies have reported variable proportions, from 5 to 60%) causing serious economic and social impacts on both the individual and the affected communities [39]. Death by CHIKV infection is rare (<1% of infected people) and occurs mostly in older adults [39].

Diagnosis is made through demonstration of CHIKV IgM antibody in the serum and also by reverse transcriptase-polymerase chain reaction (RT-PCR) from ocular fluids and serum [40].

There is no specific antiviral drug treatment for CHIKV; therefore treatment is directed primarily for relieving the symptoms, including the joint pain and fever, using anti-pyretics, optimal analgesics and fluids [41, 42].

### Ocular disease

The ocular manifestations are not frequent and can range from anterior to posterior segment involvement. Although the lack of publications in this area there are few reports demonstrating that the most common symptoms are photophobia, retrobulbar orbital pain, and conjunctivitis. The main ocular manifestation is an anterior uveitis, often associated with pigmented keratic precipitates and ocular hypertension [43, 44]. Other anterior segment manifestations had been described as such scleritis, keratitis, nerve palsy, nystagmus and myositis [45].

The management of the anterior uveitis evolves topical and systemic corticosteroids to control inflammation [43, 44].

### Retinal manifestations

**Fig. 3:**
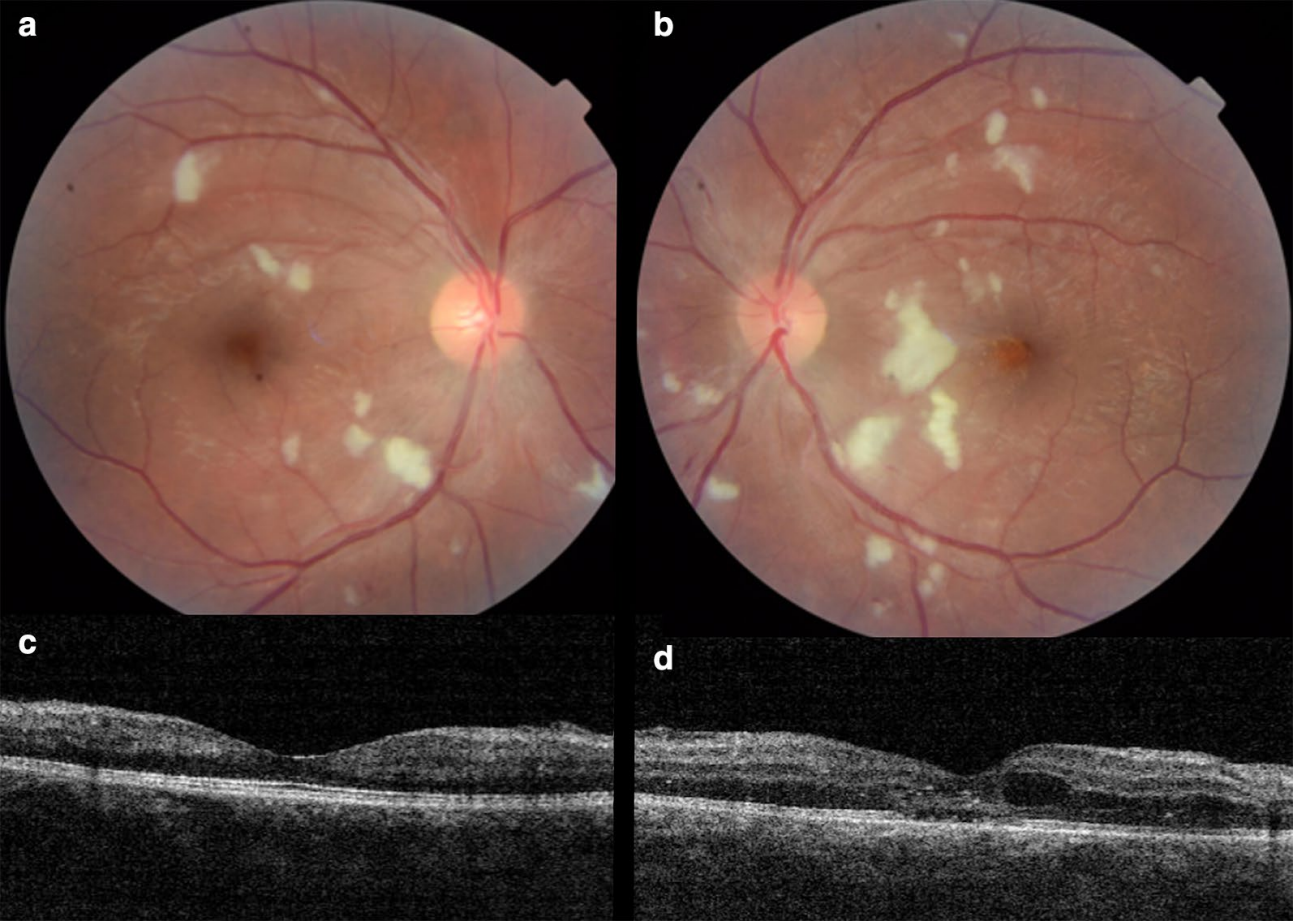
Fundus photographs of the right (**a**) and left (**b**) eyes of a 28 years old male patient with presumed retinitis secondary to Chikungunya virus infection reveals cotton-wool spots in both eyes and retinal hemorrhage and macular edema in the left eye. The horizontal B-scan of OCT on macular area shows no significant alterations in the right eye (**c**) and intra-retinal fluid, intra-retinal hyperreflective points correspondent to exudates and focal loss of external limitant membrane and ellipsoid zone of the left eye (**d**)

Posterior segment involvement of CHIKV infection may manifest as choroiditis, retinitis, neuroretinitis and optic disc neuritis [43, 44]. Chikungunya retinitis (CR) can present at the time of fever or after many weeks or months of the infection. Clinical features include vitritis, hyperemic disc, retina hemorrhages, cotton wool spots (Fig. 3) and multifocal retinitis [44, 45].

The main differential diagnoses are herpetic and cytomegalovirus retinitis but both of them are more common in immunodeficient individuals with a more exuberant inflammation. Although CR may morphologically mimic herpetic or cytomegalovirus retinitis, the history of fever, joint pains, and skin rash before the onset of visual symptoms is helpful in the diagnosis, particularly in the endemic regions [45].

## Conclusions

Arboviruses are transmitted by arthropods, including an alphavirus (CHIKV) and flaviviruses (DFV and ZIKV). The main vectors are *A. aegypti* and *A. albopictus.* The prevalence of these arboviruses has increased in the past few years, as also the incidence of systemic and ocular manifestations has been better understood. Ocular manifestations of DFV, ZIKV and CHIKV infections can be present at the time of fever or may manifest after many weeks. Anterior uveitis, optic neuritis, and retinitis are the most common manifestations during the acute infection.

The exact pathogenesis of the ocular manifestations, development of specific antiviral therapy, and vaccination against these three arboviruses are fields that require further research. Efforts to eradicate mosquitos are critical and could also reduce infection rates with other mosquito-borne illnesses such as DFV, ZIKV, and CHIKV. These efforts are already under way globally and rely primarily on environmental modifications. Genetic manipulation of mosquito populations, such as the recently described gene-drive system that can introduce female sterility into a target vector population, may enhance these efforts.
